# Insights from 40 years of educational research: honoring Jeroen van Merriënboer

**DOI:** 10.1186/s41077-025-00332-8

**Published:** 2025-03-13

**Authors:** Jimmy Frèrejean, Katie Walker, Ben Symon, Walter Eppich

**Affiliations:** 1 School of Health Professions Education, Department of Educational Development and Research, Faculty of Health, Medicine and Life Sciences, Maastricht University, Maastricht, The Netherlands; 2 Mater Education, Mater Misericordiae Limited, South Brisbane, QLD Australia; 3 School of Clinical Medicine, University of Queensland, Brisbane, QLD Australia; 4 Collaborative Practice Centre and Department of Medical Education, Faculty of Medicine, Dentistry and Health Sciences, University of Melbourne, Melbourne, Australia

**Keywords:** Simulation-based education, Instructional design, 4C/ID, Educational science, Transfer of learning

## Abstract

Simulation-based education in healthcare has advanced significantly, yet a persistent gap remains between educational science and healthcare simulation research. The late Jeroen van Merriënboer’s extensive work in educational science provides valuable guidance for bridging this gap. Four key insights from his research can serve as a strong theoretical bedrock for educators and researchers aiming to design more effective and cohesive simulation-based learning experiences: (1) integrating learning in both simulated and real environments to improve transfer, (2) offering targeted learner support that evolves with expertise, (3) embracing the complexity of educational practice and avoiding one-size-fits-all solutions, and (4) embedding domain-general skills within specific disciplines. Championing these insights may catalyze more theory-informed practice and research in healthcare simulation. Nevertheless, applying these principles in practice remains a challenge, highlighting the need for further research into the “how”—specifically how to interconnect learning environments, adapt instruction to diverse needs, integrate theory with practice, and combine the teaching of domain-general and domain-specific skills.

## Introduction

In recent years, healthcare simulation-based education (SBE) has witnessed remarkable advancements driven by technological innovation, accompanied by a growing recognition of the need for practical, hands-on training to prepare health professionals for complex and often team-based patient care. A range of studies document the benefits of SBE in improving clinical and team-based skills, leading to better patient outcomes [1–4]. However, despite these advancements, we see a persistent disconnect between the rich insights published in the educational science and educational psychology literature and those found in healthcare simulation journals. This disconnect can result in parallel but separate spheres of knowledge and contribute to a fragmentation of simulation research. The 2023 Society for Simulation in Healthcare Research Consensus Conference revealed that much of the published research in healthcare simulation is of varying quality and heterogeneous, complicating meaningful synthesis [5]. As McGaghie and Webster noted, “Scattered, one-shot, disconnected studies are less likely to inform best practices in health science education than investigations that contribute to a thematic research line” ([6] p. 587). These issues underscore an urgent need for theoretically grounded, thematic, and cumulative scholarship focusing on interrelated topics that build upon each other over time. Such scholarship amplifies its impact and yields enduring changes in educational practices. Numerous calls in the health professions education literature echo this [5, 7, 8].

Addressing these challenges requires drawing on scholars whose work bridges disciplinary divides and fosters coherent, programmatic research. Jeroen van Merriënboer (1959–2023) exemplified such a scholar. Van Merriënboer was a Professor of Learning and Instruction at the Department of Educational Development and Research and research director at the School of Health Professions Education at Maastricht University in the Netherlands. Over his 40-year career of sustained, thematic, and cumulative work, he contributed to educational science, instructional design, and health professions education. He published more than 450 scientific papers and book chapters. Although his work has influenced many health professionals and researchers, we believe there is considerable room for more explicit uptake within the healthcare simulation community. Van Merriënboer’s contributions to cognitive load theory (CLT) revolutionized our understanding of how learners use cognitive resources during learning and how instructional design can optimize these processes [9, 10]. CLT describes how educators can manage the cognitive load to enhance learning outcomes [11, 12] and increasingly influences current simulation and debriefing practices [13, 14]. Van Merriënboer also developed the Four-Component Instructional Design (4C/ID) model [15–17]. This model focuses on designing training for complex learning by integrating four components: learning tasks, supportive information, procedural information, and part-task practice. It strongly emphasizes simulation-based learning and presents numerous guidelines for designing comprehensive learning programs. Despite their profound relevance, Van Merriënboer’s recommendations for instructional design have thus far found only limited traction in healthcare SBE [18, 19].

At his retirement, Van Merriënboer delivered an inspirational farewell lecture entitled *Learning in Real and Simulated Learning Environments* [20], in which he shared four key insights gained from a 40-year research career. The critical learnings he developed deeply impacted this author team: JF, KW, and WE. We have benefited from Van Merriënboer’s example and sage guidance in our research journeys. Given the exemplary nature of his significant body of work for our current practice of healthcare simulation, we feel compelled to disseminate a focused summary of his four insights and their potential impact on healthcare simulation and health professions education. This article aims to bridge the gap between educational science and healthcare simulation science by highlighting Van Merriënboer’s contributions as the theoretical bedrock for educators to build more effective and cohesive simulation-based learning experiences. The following sections describe each insight in Van Merriënboer’s Farewell Lecture [20], starting with a summary in italics followed by a brief discussion of implications for the field of healthcare simulation.

## Insight 1: Reality

### Summary

**Fig. 1:**
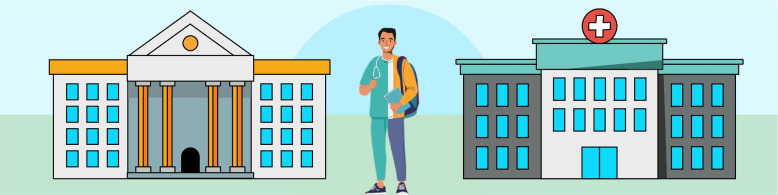
Learning in the educational institute and the workplace should be strongly interconnected to optimize learning

*Van Merriënboer’s first insight emphasizes linking educational experiences closely with real-world practice. Van Merriënboer discovered that traditional teaching methods often result in fragmented knowledge, where learners learn isolated skills without being able to apply them effectively in real situations. To address this fragmentation, he advocated for designing learning tasks that mirror professional tasks, ensuring learners engage with realistic, meaningful challenges from the start of their learning program. This approach is especially effective in vocational and professional education, such as healthcare, where combining simulated scenarios with real clinical practice helps learners transfer skills more effectively to the workplace. This reciprocal connection between learning in simulated and real-world settings enhances the overall learning experience and better prepares learners for professional tasks* (Fig. 1).

Simulation in healthcare education is a powerful tool to bridge the gap between academic learning and clinical practice. However, its effectiveness is maximized when it is part of a comprehensive learning program that also includes workplace experiences. Rather than treating simulations as isolated events, educators should view them as integral components of a continuous learning loop, where simulation and workplace practice reinforce each other [21]. Realizing this integrated vision might require a paradigm shift, as the prevailing culture in health professions education often emphasizes *training* skills before *applying* them in practice rather than blending learning across formal training and practice. Programs that integrate simulation and real-world practice from the outset can create a smoother transition to the workplace, and alleviate the abrupt shift that can occur when the two are treated separately [22].

Strengthening the interconnectedness between simulation and workplace learning can be done in several ways. For example, *learners* can bring or select simulation scenarios relevant to their current learning needs instead of practicing scenarios selected by the *educator* [23]. Alternatively, educators can conduct needs assessments to gather input from learners about difficulties they face in their clinical practice, ensuring that simulations address real-world issues and are more impactful. Post-simulation debriefings can connect the experience to these challenges [24]. In addition to promoting reflection, debriefings can encourage learners to think ahead and discuss how they will apply their learning in future clinical tasks. A final example includes assigning supervised tasks in the clinical setting that directly relate to the skills practiced in simulation. For instance, after a simulation on emergency airway management, learners might be paired with an experienced clinician to practice or observe airway procedures in the actual clinical environment soon afterward, reinforcing new competencies. Job aids or workplace supports, such as checklists or brief guidelines, can be pinned in relevant clinical workspaces to ensure that what is learned in simulation is not forgotten once back on duty. When simulation is closely tied to clinical practice, it leads to deeper learning and better preparation for real-world challenges.

A second implication of this insight is that simulations should mirror the complexity and authenticity of real-life clinical situations. However, this does not mean defaulting to high-tech simulators in every case. Instead, following Van Merriënboer’s perspective, the focus should be on ensuring that simulations engage learners in the same cognitive processes required for real-world tasks. How closely a simulation resembles reality in appearance, sound, or feel can be adjusted depending on the learning objectives [15]. In addition, part-task simulations—where learners focus on specific skills or procedures, such as suturing or intubation—might be valuable for building foundational skills, but should not be considered sufficient on their own. One of the critical insights from educational models like the 4C/ID model is that learning isolated components does not automatically translate into competent whole-task performance. For instance, learners may master isolated skills like inserting an intravenous catheter or performing cardiopulmonary resuscitation in a simulation [25]. However, without practice integrating these skills into a full clinical scenario—such as managing a patient with multiple trauma in an emergency room—they may struggle to apply them effectively in real situations. Whole-task practice, where learners work through entire clinical cases that require integrating various skills, is necessary to develop this ability [26, 27]. By consistently pairing part-task practice with whole-task practice and avoiding isolated skill training, we improve the likelihood of learning transfer [28].

## Insight 2: Learner support

### Summary

**Fig. 2:**
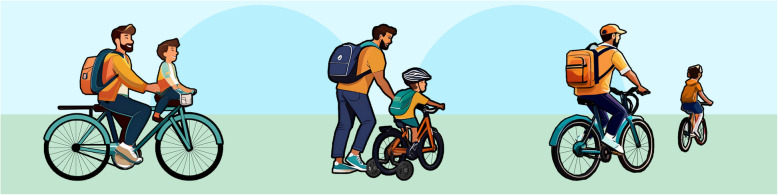
To optimize learning in simulated and real environments, it is critical to provide the right type and amount of support to learners—not too little and not too much

*Van Merriënboer’s second key insight emphasizes the critical role of providing support to learners. He observed that merely performing a task does not guarantee learning. Learners need targeted support, such as theoretical background information, just-in-time instruction, feedback, and guidance. However, the effectiveness of this support depends on various factors, including the learner’s prior knowledge, learning goals, and current conditions. For instance, worked examples can be highly effective for beginners but may hinder more experienced learners, a phenomenon called the “expertise reversal effect.” As learners gain more knowledge, the type and amount of support should evolve, gradually decreasing in a process known as scaffolding. Furthermore, support should ideally be tailored to individual needs, considering cognitive factors and emotional and motivational aspects to optimize learning. This tailored approach ensures that learners receive the right kind of help at the right time, enabling them to tackle challenging tasks and achieve their learning goals successfully* (Fig. 2).

Simply guiding learners through a simulation scenario is often insufficient to ensure deep learning. Although experiential learning—where learners learn by doing—is frequently used as underpinning and justification for SBE [29, 30], it alone does not guarantee that learners will fully grasp and retain the necessary skills and knowledge. A more comprehensive approach integrates simulations with other instructional strategies that support and deepen learning. For example, learners will often acquire theoretical background through readings, e-learnings, group discussions, or case studies that help them understand the prerequisite knowledge and skills relevant to the simulation practice. These study activities can precede the simulation or emerge from post-simulation feedback, fostering repeated cycles of theory, practice, and workplace application rather than prolonged stretches of a single approach. Educational research highlights the importance of curriculum integration [31].

It is important not to confuse *goals*, such as learning teamwork or problem solving, with *methods*, such as simply placing learners in teams or giving them problems to solve. When training complex skills, Van Merriënboer recommends presenting worked-out examples, modeling examples, or demonstrations so learners can first study how experts approach tasks rather than engaging in simulations from the beginning. For instance, an instructor might “think aloud” while demonstrating a clinical procedure, providing real-time insight into expert decision making; learners could study a 360-degree video of an authentic task demonstrating good teamwork in its actual environment; or they could analyze a written case study that captures the nuances of expert clinical reasoning. After studying examples, learners gradually transition to guided practice with simulations. Educators can then use just-in-time teaching—offering immediate guidance or clarification when learners encounter difficulties—to steer them in the right direction without taking over the task. After the simulation, debriefings reinforce learning. Focusing these debriefing sessions on what happened during the simulation and the theory and examples provided earlier will help learners see how everything fits together. Constructive feedback is essential, allowing learners to understand what they did well and where they can improve and to make connections between their performance in the simulation and the real-life clinical situations they will face. This approach of integrating simulations with other learning activities, known as simulation-enhanced learning [28], goes beyond simply putting learners into realistic scenarios. It regards simulations as part of a larger educational strategy that also includes methods aimed at study, real-time support, and thorough reflection.

Providing scaffolding in SBE is a complex but fundamental task. Scaffolding refers to the support educators provide to learners. Selecting the right combination of tools to offer this support is challenging because there is no one-size-fits-all approach [32]. Each learner has unique needs, competencies, and preferences, meaning the level and type of support must be tailored to their circumstances. For some learners, the key is introducing additional challenges or *desirable difficulties* [33] that push them to stretch their capabilities and stimulate deeper learning. For others, it might be necessary to increase the level of support to help them complete the task. One common pitfall in simulation design is creating overly complex scenarios that, while possible in theory, are not realistic or conducive to meaningful learning. These scenarios can overwhelm learners, making focusing on the critical learning objectives difficult. A consistent aim should be to design challenging yet manageable scenarios without *overloading* or *underloading* a learner’s cognitive capacity [34]. To aid in this process, educators could utilize scenario databases that offer a range of scenarios with varying levels of support, complexity, and content. By selecting scenarios that align with a learner’s specific learning needs, educators can better match the level of challenge to the learner’s current abilities and sequence subsequent scenarios in a way that gradually reduces the level of scaffolding as learners develop their skills.

The challenge of scaffolding becomes even more pronounced in group simulations, especially in interprofessional education settings where learners from different disciplines come together with diverse learning goals. Analyzing individual learning needs and carefully designing scenarios that accommodate these varying goals as much as possible remains essential in these cases. Educators should be skilled in offering real-time support and feedback during simulations, adjusting the level of assistance to ensure every learner gets the most out of the experience.

## Insight 3: Complexity

### Summary

**Fig. 3:**
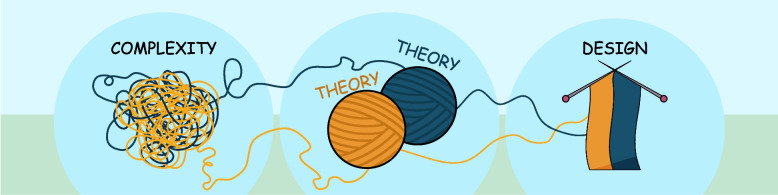
We need empirically supported educational theories to help us understand teaching and learning processes and make design decisions to optimize learning in simulated and real environments

*The third insight highlights the inherent complexity of educational research and practice. Unlike more straightforward sciences, where clear-cut answers might be found, education is filled with “ubiquitous interactions” that make it difficult to declare any educational method universally effective. There are no “good” or “bad” instructional methods; each method’s effectiveness depends on the specific learning context, goals, and conditions. This complexity challenges the notion of evidence-based education, which suggests that specific methods are universally effective based on empirical evidence. Educational research should instead focus on developing theories that explain how and why certain methods work in specific contexts. These theories should guide educators in making informed decisions based on problem solving, reasoning, and applying deep understanding rather than following a simplistic, one-size-fits-all approach. Thus, instructional designs should be grounded in scientific theory, supported by empirical findings, and used to inform practice rather than relying on rigid “cookbook” methods* (Fig. 3).

Teaching healthcare professionals through simulation is inherently complex, requiring flexibility and adaptability. Education is nuanced; what works in one situation may not work in another. For example, a study might suggest that a simulation approach using rapid-cycle deliberate practice improves performance in pediatric residents [35]. While this may be true for a particular group of learners in a specific setting, it does not mean that rapid-cycle deliberate practice will work in every context and for other learning goals [36]. Rather than rigidly applying methods from studies or other institutions, educators must develop a deep understanding of how different instructional methods function in various contexts. The key is to stay flexible, adapting the design based on insights from both experience and ongoing research. Effective SBE is not about a one-size-fits-all recipe but about continuously refining methods, understanding learners’ unique needs, and adjusting strategies for the most impactful learning experiences.

Van Merriënboer’s 4C/ID model offers a comprehensive framework that explains why certain instructional methods work effectively in specific situations. This model is highly relevant for simulation educators because it provides a structured framework for designing learning experiences that replicate real-world complexity while ensuring learners receive the necessary support, guidance, and feedback to develop complex skills [37]. It was specifically designed to promote reflective expertise [38], commonly called “adaptive expertise” today [39, 40]. The 4C/ID model integrates four key components: (1) engaging learners in whole-task practice in real and simulated environments, (2) providing necessary supportive information, (3) providing just-in-time procedural information, and (4) incorporating part-task practice when needed. The model is grounded in scientific research from different domains, offering clear guidelines for effectively implementing each component [41, 42]. Understanding and applying such theories is essential for those designing or researching SBE, yet adherence to instructional design guidelines seems poor [18, 19]. Theories help ensure that the methods educators choose are not based on intuitive ideas but theory-based and carefully tailored to fit learners’ specific learning needs and contexts [43].

Researchers in SBE play a vital role in advancing the field by contributing to the development of instructional theories. For individual intervention studies to contribute to theory-building, they must go beyond reporting whether a particular method worked or did not work. Researchers must carefully document the specific context in which the research was conducted, including detailed characteristics of the learners and environment (e.g., confounding variables), well-defined learning objectives (e.g., conditions, behavior, standard of performance), evidence of formative evaluations, and a clear description of which instructional methods were combined or compared [44, 45]. Furthermore, reporting of outcomes should go beyond effectiveness and instead describe the trade-off between the “iron triangle” of effectiveness, efficiency, and enjoyability [46]. Omitting any of these three outcomes conceals the trade-offs from particular design decisions and makes comparisons across studies far less meaningful. This detailed contextualization is crucial for refining theories that explain which methods work under which circumstances. By designing studies that contribute to a cumulative body of knowledge rather than produce isolated findings, researchers can enhance understanding of how different instructional methods perform in diverse contexts and with different learners. In this light, meta-analyses that examine instructional methods without considering learners, goals, or contexts compare apples to oranges and offer limited insights despite a veneer of an evidence base.

## Insight 4: Domain-general skills

### Summary

**Fig. 4:**
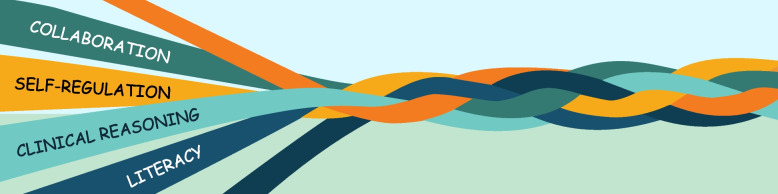
To optimize learning in simulated and real environments, the instruction of domain-general skills should be closely intertwined with domain-specific skills

*The fourth insight addresses the complexity of teaching domain-general skills—those skills that are applicable across various fields, such as problem solving, collaboration, self-regulation, and information literacy. Although these skills are widely recognized as essential for twenty-first-century learning, a key challenge is that they cannot be taught effectively in isolation. Domain-general skills must be integrated with domain-specific skills, meaning they should be taught within the context of a specific subject or field. For example, problem solving in healthcare differs fundamentally from problem solving in mathematics; thus, teaching general problem-solving skills outside a particular domain is unlikely to be effective. This intertwined approach has significant implications for both learner support and curriculum design. Educators need to provide support on two levels: domain-specific (first-order scaffolding), such as offering direct resources or information, and domain-general (second-order scaffolding), such as teaching learners how to find and use resources independently. Additionally, curriculum designers should ensure that domain-general skills are embedded within subject-specific courses rather than taught separately* (Fig. 4).

As healthcare education evolves, many programs increasingly adopt competency-based approaches requiring learners to regulate their learning [47]. This shift places a significant responsibility on learners to select their learning tasks, find appropriate study materials, and engage in self-directed practice. However, this level of autonomy demands a set of complex skills that need to be explicitly developed rather than assumed. Simulation educators must recognize that learners may need guidance in developing these skills, particularly in a healthcare context with high stakes and steep learning curves [48]. One key area where this is evident is the development of information literacy—locating, evaluating, and effectively using relevant data and resources—and evidence-based practice, which entails critically appraising current scientific findings and integrating them with clinical expertise to guide patient care. Traditionally, these skills might be taught in isolated courses or workshops. However, research suggests the inadequacy of this approach [49]. Instead, educators should strive to integrate the teaching of these skills directly into domain-specific courses. For example, rather than teaching evidence-based practice as a standalone course, it could be woven into a course that includes simulations. Here, learners might be tasked with finding and appraising relevant research to prepare for a simulation scenario. Support and feedback can then target the performance in the scenario (first order) as well as the search strategy used to retrieve evidence (second order) and fade out as learners gain competence. This integration helps learners see the direct relevance of literacy skills to their professional practice and reinforces their application in a realistic context.

A practical application of this integrated approach is in the use of independent part-task practice. Part-task simulators allow learners to focus on specific task components, such as suturing a wound or inserting an intravenous catheter, without the complexity of a full-scale simulation. For this self-directed practice to be effective, learners must be taught how to use these simulators properly. The prerequisites for self-directed learning extend beyond simply demonstrating the simulator’s technical aspects. They include teaching learners how to set goals, emphasizing the importance of distributed practice and setting appropriate practice intervals, helping them use feedback to refine their skills, and explaining why part-task practice should be alternated with whole-task practice. To truly support learners in a competency-based education framework, simulation educators must do more than provide access to tools and resources. They must actively teach self-regulated [50] and self-directed learning skills [51] to ensure learners can manage their professional development.

## Conclusions

Van Merriënboer’s extensive research thoroughly explores these four essential insights—reality, learner support, complexity, and domain-general skills—that carry profound implications for SBE. First, it underscores the importance of integrating learning across both simulated and real environments, ensuring simulation-based learning is directly applicable to clinical practice. Second, it highlights the need for tailored support that adapts to individual learners and emphasizes the role of scaffolding in guiding them through increasingly complex tasks. Third, it recognizes the inherent complexity of teaching and learning, urging educators to move beyond one-size-fits-all approaches and to embrace flexibility and adaptability in their theory-informed instructional strategies. Finally, it stresses the importance of teaching domain-general skills within the context of specific disciplines, especially in competency-based programs where learners are expected to self-regulate their learning. We have offered examples of how these insights might be applied in clinical settings, hopefully catalyzing future research and discussion. Van Merriënboer acknowledges that his insights are not new or original, and while they describe *what* should be done, they do not necessarily explain *how*. He states: “We know surprisingly little about the how-questions: How to interconnect learning in real and simulated environments? How to adapt instruction to a broad set of learning needs? How to connect educational theory and practice? And how to intertwine the teaching of domain-specific and domain-general skills?” ([20] p.6). These “how-questions” offer fertile ground for health professions education researchers to explore further.

We hope this article has both honored Van Merriënboer’s contributions and inspired the development of more theoretically grounded and connected research in health professions education. In closing, we return to Van Merriënboer’s wise words: “The more I learned about education, the more I became aware of how little we really understand about teaching and learning. To further develop our understanding, much more research and, eventually, much more educational theory is needed” ([20] p.6). As we reflect on how much remains to be understood, let this be a rallying cry for our community to intensify efforts in bridging the gap between educational science and healthcare simulation.

## Data Availability

No datasets were generated or analysed during the current study.
